# Anesthetic management of ventricular-peritoneal shunt implantation in osteogenesis imperfecta type IIB: A case report

**DOI:** 10.1097/MD.0000000000028483

**Published:** 2022-01-07

**Authors:** Mayumi To, Kota Kamizato, Hayato Shinzato, Manabu Kakinohana

**Affiliations:** Department of Anesthesiology, Faculty of Medicine, University of the Ryukyus, 207 Uehara, Nishihara-cho, Okinawa, Japan.

**Keywords:** case report, general anesthesia, osteogenesis imperfecta

## Abstract

**Introduction:**

Osteogenesis imperfecta (OI), a rare congenital disorder, has a risk of bone fracture and progressive bone deformity. OI type II is the most serious subtype, and very few reports on its anesthetic management exist. Patients face several anesthetic difficulties, of which easy fracturing of OI-affected bones is critical. Herein, we report our experience with the anesthetic management of a patient with OI type II.

**Patient concerns and diagnoses:**

Through genetic testing, a 14-month-old girl (height: 45 cm, weight: 3.9 kg) was diagnosed with OI type IIB due to *COL1A* gene abnormality. The clinical manifestations included hydrocephalus, blue sclera, dental dysplasia, short stature, limb deformity and shortening, thoracic hypoplasia, rabbit eye, left inguinal hernia, and tricuspid valve regurgitation. Physical examination revealed an enlarged head due to skull dysplasia and hydrocephalus. The pediatrician confirmed that mask ventilation was possible even under spontaneous breathing, and that there was no history of bone fracture during mask holding.

**Interventions and outcomes:**

The patient was scheduled for gastrostomy and ventriculo-peritoneal shunt implantation. An arterial pressure line was inserted at the neonatal intensive care unit. Propofol and remifentanil were selected for general anesthesia. Confirming that mask-assisted ventilation was possible under sleep, rocuronium was administered. Attentive mask ventilation was performed via the 2-person method to avoid fractures. We were able to intubate successfully using a Macintosh laryngoscope. A microcuff endotracheal tube was used. For ventilation, pressure control ventilation was selected and sedative dosage was adjusted using the patient state index as an indicator. The patient was sedated, intubated, and returned to the neonatal intensive care unit. She was extubated on the sixth postoperative day. No bone fractures were noted.

**Conclusion:**

OI type II is the most severe subtype with high mortality, and there are few reports on its anesthetic management. Easy fractures can be a problem in airway maintenance, blood pressure measurement, and repositioning. We performed the procedure attentively, avoiding jaw and cervical fractures during mask ventilation and endotracheal intubation. For respiratory management, we chose pressure control ventilation using a cuffed tracheal tube and circulatory control was attained via an arterial line inserted preoperatively. No complications occurred.

## 1 Introduction

Osteogenesis imperfecta (OI) is a rare congenital disorder that results from an abnormality in the genes involved in type 1 collagen maturation, resulting in the increased risk of bone fracture and progressive bone deformity.^[1,2]^ It also presents with a variety of connective tissue abnormalities.

Several problems are associated with the anesthetic management of OI. First, OI-affected bones are easily fractured, which necessitates caution when maintaining the airway or performing non-invasive arterial measurements. Second, maintaining the airway may be difficult. Third, the body temperature may increase due to hypermetabolism. Among the various types of OI, OI type II is the most serious subtype, and there are very few reports on its anesthetic management.^[3]^

Herein, we report our experience with the anesthetic management of ventriculo-peritoneal shunt implantation surgery for hydrocephalus in a patient with OI type II.

## 2 Case

Through genetic testing, a 14-month-old girl (height: 45 cm, weight: 3.9 kg) was diagnosed with OI type IIB due to *COL1A* gene abnormality. The clinical manifestations included hydrocephalus, blue sclera, dental dysplasia, short stature, limb deformity and shortening, thoracic hypoplasia, rabbit eye, left inguinal hernia, and tricuspid valve regurgitation. Crying and limb movements were noted. However, no utterance was observed. Her respiratory status was normal, with an inspiratory oxygen saturation of 35% to 45% under continuous positive airway pressure. The percutaneous arterial oxygen saturation (SpO_2_) was 96% to 98%. Presurgical chest computed tomography revealed atelectasis.

The patient was scheduled for gastrostomy and ventriculo-peritoneal shunt implantation for the hydrocephalus. Physical examination revealed an enlarged head due to skull dysplasia and hydrocephalus. The patient had a large tongue; however, she was able to open her mouth enough for a laryngoscope to be inserted. The pediatrician confirmed that mask ventilation was possible even under spontaneous breathing and that there was no history of bone fracture during mask holding. Her blood pressure was not measured because of the risk of bone fracture.

The patient was admitted to the operating room without any premedication. An arterial pressure line was inserted at the neonatal intensive care unit (NICU) before entering the operating room.

Total intravenous (IV) anesthesia with propofol and remifentanil was selected for the general anesthesia. Anesthesia induction was started with propofol 60 mg/h continuous IV infusion and remifentanil 0.3 mcg/kg/min continuous IV infusion. Confirming that mask-assisted ventilation was possible under asleep and spontaneous breathing, rocuronium 0.6 mg/kg was administered intravenously. Attentive mask ventilation was performed via the 2-person method to avoid fractures during airway maintenance. Tracheal intubation was attempted by a pediatric physician using an airway scope (AWS-S200NK, Nihon Kohden, Japan). The airway scope was inserted in a patronizing manner, but the glottis could not be confirmed because of the large amount of secretions, making it difficult to secure a field of view. After changing to a Macintosh laryngoscope (G-Macintosh blade #1), we were able to intubate successfully. A microcuff endotracheal tube was used (I.D.3.5 mm, Avanos Medical, Inc., GA), and its position was confirmed by chest radiography, with the tip being located just above the tracheal bifurcation. During our multiple intubation attempts, the ventilation gradually became difficult, the SpO_2_ decreased from 100% to 25%, and the pulse rate decreased from 180 to 75 bpm. Therefore, mask ventilation with 100% oxygen and intravenous atropine 0.005 mg were administered, and intubation was performed after recovery.

The ventilator settings were as follows: pressure control ventilation, inspiratory pressure of 25 cmH_2_O, respiratory rate of 35, inspiratory-to-expiratory ratio of 1:2, and positive end-expiratory pressure of 3 cmH_2_O. These were maintained at a tidal volume of 35 to 40 mL, minute volume of 1.45 to 1.55 L/min, and an end-tidal carbon dioxide of 40 to 42 Torr.

A SedLine monitor (Masimo Corporation, CA) was attached, and the sedative dosage was adjusted using patient state index as an indicator. Specifically, propofol was tapered using the step-down method; induction was started at 60 mg/h and eventually maintained at 36 to 40 mg/h. The PSI values remained at 20 to 25. Remifentanil was maintained at 0.4 to 0.5 mcg/kg/min. The inspiratory oxygen saturation was maintained at 50%, and the SpO_2_ did not drop during surgery.

The body temperature was measured using a rectal probe. The patient's temperature before admission was 39 °C, but it improved to 35.3 °C before the surgical procedure. The extremities were heated with a warming blanket. The temperature dropped to 34.1 °C but improved to 34.7 °C at the end of the surgery.

The surgical procedure took 2 hours 15 minutes, and the anesthesia time was 4 hours 50 minutes.

The patient was sedated with propofol, intubated, and returned to the NICU. The patient was not extubated in the surgery room and continued to be ventilated in the NICU. After returning to the NICU, the patient was switched to midazolam for sedation. She was extubated on the sixth postoperative day. No bone fractures associated with the anesthetic management, or the surgical procedure were noted.

The patient and her parents provided written informed consent for the publication of this case report.

## 3 Discussion

Although Sillence classification^[4]^ is used in clinical settings, OI is a genetically heterogeneous syndrome associated with various genetic abnormalities.^[5]^ Its incidence is approximately 1 case per 10,000 to 20,000 births.^[1,2]^ In patients with OI, the bone tissues are characterized by a decreased bone mass and increased bone density. Decreased bone strength leads to skeletal deformities, resulting in low-trauma bone fractures and fractures in atypical locations.^[6]^

Non-skeletal symptoms include dental abnormalities, blue-gray sclera, hearing loss, decreased joint mobility, rare muscle weakness, and cardiovascular and pulmonary complications.^[2,6]^ In this case, the clinical symptoms included hydrocephalus as a central nervous system symptom, hypoplasia of the thorax and tricuspid valve regurgitation, limb deformity and shortening, and a short stature. Given these, respiratory and circulatory management required special attention.

In our patient, the issues during anesthetic management included easy bone fracture, body temperature control, congenital valve disease, and respiratory management associated with thoracic hypoplasia.^[7]^ Each abnormality has been reported to vary greatly in severity depending on the type of OI.^[1,2]^ OI type II is the most severe and the number of survivors is small, and there are few reports on their anesthetic management.

Easy fractures can be a problem in airway maintenance, blood pressure measurement, and repositioning. In this case, we performed the procedure attentively, taking care to avoid jaw and cervical fractures during mask ventilation and endotracheal intubation. Mask ventilation was performed using the 2-person method, and an airway scope was planned for intubation. However, because the patient had a large tongue and increased secretions, the intubation view with the Airway Scope was poor, prompting us to switch to a G-Macintosh laryngoscope for intubation. The multiple intubation attempts resulted in edema of the vocal cords, which made mask ventilation difficult.

Moreover, the possibility of bone fracture due to cuff pressure during non-invasive blood pressure measurements has been reported. Therefore, management with an arterial pressure line may be effective in reducing the risk. Conversely, in an observational study on the surgical management of OI, no complications were observed despite the use of non-invasive blood pressure measurement.^[8,9]^ They also reported no fractures after using a 250-mm Hg tourniquet.^[9]^ However, this study did not include OI Type II cases. In this case, after conferring with the pediatrician, we decided to secure an arterial pressure line preoperatively to enable the monitoring of her blood pressure.

For respiratory management, our patient's respiratory setting might have been difficult if compliance decreased due to thoracic deformities. Especially in pediatric patients, it is assumed that ventilation cannot be maintained when a cuffless tube is used. In this case, we managed the patient using a cuffed tracheal tube (microcuff endotracheal tube ID 3.5 mm) in consideration of the possibility of decreased compliance. Consequently, the compliance after the introduction was 2 mL/H_2_O, and the maximum airway pressure was 26 to 28 cmH_2_O, which did not exceed 30 cmH_2_O, making respiratory management possible.

Patients with OI are believed to be prone to intraoperative hyperthermia due to hypermetabolism.^[7,10]^ However, some reports suggest that these perioperative temperature changes are similar to those in patients without OI and that patients with OI are not particularly prone to hyperthermia.^[11]^ In our case, the patient had a high body temperature before surgery, with an axillary temperature in the range of 39.0 to 39.5 °C. In the NICU, the patient's body temperature was controlled by cooling. At the start of the surgical procedure, the rectal temperature was 35.3 °C, indicating hypothermia. Although heating was started with a warming blanket, the intraoperative body temperature did not increase, and the rectal temperature remained at 34.1 to 34.5 °C. In the operating room, continuous temperature measurements and temperature control should be initiated immediately.

In conclusion, we experienced the perioperative management of the most severe form of OI, Type IIB. Total intravenous anesthesia with propofol and remifentanil was administered. Tracheal intubation was used for airway management, and circulatory control was attained via an arterial line inserted preoperatively. No complications occurred.

## Acknowledgments

The authors would like to thank Editage (www.editage.com) for English language editing.

## Author contributions

**Conceptualization:** Manabu Kakinohana.

**Writing – original draft:** Mayumi To, Kota Kamizato.

**Writing – review & editing:** Hayato Shinzato, Manabu Kakinohana.
